# Effects of Elevated Temperature on *Pisum sativum* Nodule Development: I—Detailed Characteristic of Unusual Apical Senescence

**DOI:** 10.3390/ijms242417144

**Published:** 2023-12-05

**Authors:** Tatiana A. Serova, Pyotr G. Kusakin, Anna B. Kitaeva, Elena V. Seliverstova, Artemii P. Gorshkov, Daria A. Romanyuk, Vladimir A. Zhukov, Anna V. Tsyganova, Viktor E. Tsyganov

**Affiliations:** Department of Biotechnology, All-Russia Research Institute for Agricultural Microbiology, Podbelsky Chaussee 3, Pushkin 8, Saint Petersburg 196608, Russia; t.serova@arriam.ru (T.A.S.); pyotr.kusakin@arriam.ru (P.G.K.); akitaeva@arriam.ru (A.B.K.); elena306@yandex.ru (E.V.S.); a.gorshkov@arriam.ru (A.P.G.); d.romanyuk@arriam.ru (D.A.R.); vzhukov@arriam.ru (V.A.Z.); avtsyganova@arriam.ru (A.V.T.)

**Keywords:** bacteroid, elevated temperature, global warming, heat-shock proteins, infection thread, legume–rhizobial symbiosis, leghemoglobin, senescence, symbiosome

## Abstract

Despite global warming, the influence of heat on symbiotic nodules is scarcely studied. In this study, the effects of heat stress on the functioning of nodules formed by *Rhizobium leguminosarum* bv. *viciae* strain 3841 on pea (*Pisum sativum*) line SGE were analyzed. The influence of elevated temperature was analyzed at histological, ultrastructural, and transcriptional levels. As a result, an unusual apical pattern of nodule senescence was revealed. After five days of exposure, a senescence zone with degraded symbiotic structures was formed in place of the distal nitrogen fixation zone. There was downregulation of various genes, including those associated with the assimilation of fixed nitrogen and leghemoglobin. After nine days, the complete destruction of the nodules was demonstrated. It was shown that nodule recovery was possible after exposure to elevated temperature for 3 days but not after 5 days (which coincides with heat wave duration). At the same time, the exposure of plants to optimal temperature during the night leveled the negative effects. Thus, the study of the effects of elevated temperature on symbiotic nodules using a well-studied pea genotype and *Rhizobium* strain led to the discovery of a novel positional response of the nodule to heat stress.

## 1. Introduction

Humanity is currently living in an era of global climate change. According to the Intergovernmental Panel on Climate Change, an increase in greenhouse gas concentration is predicted to increase the ambient temperature by 4.4 °C by the end of this century [1]. The influence of global warming is especially pronounced in the high northern latitudes. The temperature increase is one of the main stress factors associated with global climate change for plants [2]. The possible impact of global warming on the main agricultural crops, in particular legumes capable of nitrogen fixation, may adversely affect the yield [3]. However, detailed studies of the cellular and molecular responses of symbiotic nodules to heat stress are limited [4], although the first studies on the effect of high temperature on nodule development started more than 100 years ago [5].

The pioneer studies revealed that in pea (*Pisum sativum* L.) plants grown at 30 °C, nodulation was blocked. The maximum number of nodules was formed at a temperature close to the thermal limit, while the average nodule size was significantly reduced. The authors suggested that temperature affected the efficiency of nitrogen fixation and nitrogen assimilation by plants [5]. However, only 40 years later, elevated temperature was shown to inhibit nitrogen fixation in *Trifolium subterraneum* L. [6]. Different species of legumes were shown to have different tolerances to high temperatures [7]. The optimal temperature range for the development of indeterminate nodules in legumes in temperate climates is from 15 to 25 °C. For tropical legumes with determinate nodules, the upper limits are in the range of 27 to 35 °C [8]. Tolerance to elevated temperatures also varies among legume species forming the same type of nodules. Common bean (*Phaseolus vulgaris* L.) symbiosis was more sensitive to elevated temperatures compared to the soybean (*Glycine max* (L.) Merr.) and cowpea (*Vigna unguiculata* (L.) Walp.) symbioses. The common bean formed numerous small nodules with reduced nitrogenase activity when plants were grown at elevated temperatures [9]. Axillaris (*Macrotyloma axillare* (E. Mey.) Verdc.) also formed nodules at a lower temperature (26 °C) than siratro (*Macroptilium atropurpureum* (DC.) Urb.) (31 °C) [10].

In turn, tolerance of nitrogen fixation to high temperatures might depend on the strain of rhizobia [11,12,13,14,15,16,17]. It was assumed that nitrogen fixation is a temperature-sensitive process, with effective nitrogen fixation occurring in a narrow temperature range [18]. In peas, high temperature can inhibit nodule formation, lead to a decrease in the number of nodulation sites, and alter bacterial adhesion to root hairs [19].

The structural aspects of nodule development under high-temperature conditions have been little studied. It was shown that *T. subterraneum* nodules formed by a rhizobial strain NA30 sensitive to high temperature (30 °C) were characterized by numerous abnormalities: the formation of multibacteroid symbiosomes, destruction of symbiosome membranes and degradation of bacteroids, increased branching, and subsequent disruption of infection threads [15]. In turn, the transfer of 10-day-old *T. subterraneum* plants grown at 22 °C to 30 °C led to the induction of a senescence zone at the base of the nodule after 4 days of exposure [15]. Stress-induced senescence of the nodule is caused by various abiotic factors, such as drought and salinity [20,21,22,23], darkness and inhibition of photosynthesis [24,25,26,27,28], cold [29], cadmium [30,31,32], and exogenous nitrates [23,25]. Although both natural and induced senescence are associated with the formation of a senescence zone in the base of the nodule, the former gradually expands as the nodule ages, while the latter causes more rapid degradation of the whole nodule [33].

This study revealed an unusual activation of senescence at the apical part of nodules formed with the *Rhizobium leguminosarum* bv. *viciae* 3841 strain in plants of the pea line SGE exposed to elevated temperature (28 °C). Comparative analyses of histological and ultrastructural organization of the 3841 strain nodules exposed to optimal (21 °C) and elevated (28 °C) temperatures, their transcriptomic profiles, and expression of genes associated with senescence, response to heat, and defense responses were performed. The ability of nodules to recover after transfer from 28 °C to 21 °C was studied. Finally, it was shown that the exposure of plants to optimal temperatures during the night leveled the negative effects of elevated temperatures.

## 2. Results

### 2.1. Elevated Temperature in Pea Line SGE Leads to Apical Nodule Senescence

**Figure 1:**
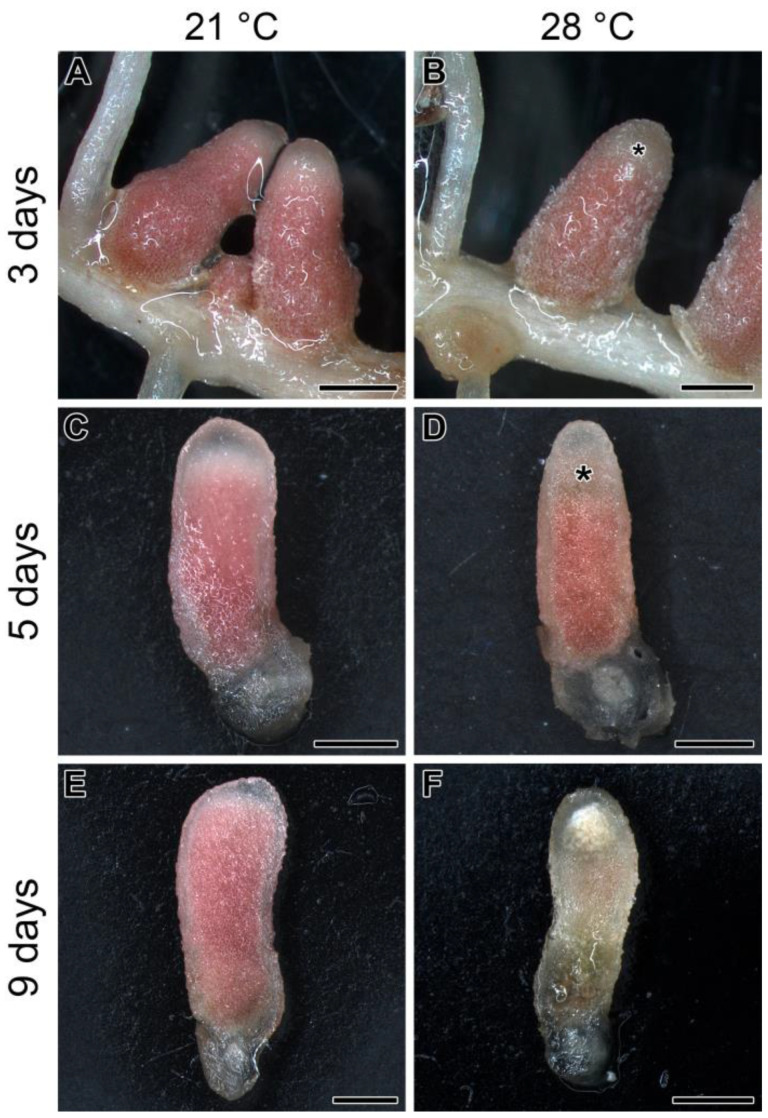
Nodules of the pea (*Pisum sativum*) line SGE in 3 (**A**,**B**), 5 (**C**,**D**), and 9 (**E**,**F**) days of exposure to 21 °C (**A**,**C**,**E**) or 28 °C (**B**,**D**,**F**). Whole (**A**,**B**) and longitudinally cut (**C**–**F**) nodules are given. Plants were inoculated with the *Rhizobium leguminosarum* bv. *viciae* 3841 strain. Asterisks indicate an emerging senescence zone in the apical part of the nodule. Scale bars are 1 mm.

When 3-week-old pea plants of the SGE line inoculated with the 3841 strain were exposed to elevated temperature (28 °C), visible effects began to be observed in the apical part of the nodule after 3 days of exposure (Figure 1A,B). After 5 days of exposure, these changes (color change from pink to greenish) were more pronounced (Figure 1C,D) and were especially pronounced after 9 days of exposure to elevated temperature (Figure 1E,F). It should be noted that nodule color change indicates senescence induction; therefore, it can be concluded that elevated temperature leads to an unusual apical pattern of nodule senescence in the line SGE.

### 2.2. Histological and Ultrastructural Organization in Heat-Stressed and Heat-Unstressed Nodules in the Line SGE

**Figure 2:**
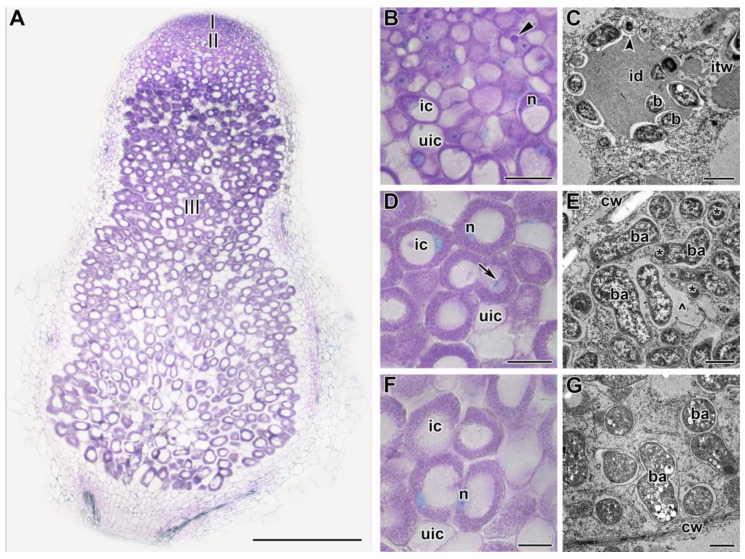
Histological and ultrastructural organization in nodules of the pea (*Pisum sativum*) line SGE in 1 day of exposure to 28 °C. (**A**) General view. (**B**,**D**,**F**) Histological and (**C**,**E**,**G**) ultrastructural organization of nodule tissue: cells from the infection (**B**,**C**), early (**D**,**E**), and late nitrogen fixation (**F**,**G**) zones. I, meristem zone; II, infection zone; III, nitrogen fixation zone. ic, infected cell; uic, uninfected cell; n, nucleus; id, infection droplet; itw, infection thread wall; cw, cell wall; b, bacterium; ba, bacteroid; *, spherical inclusion in the bacteroid; ^, multibacteroid symbiosome. The arrow indicates an infection thread, triangle indicates infection droplet, and the arrowhead indicates a degraded released bacterium. Histological sections were stained with toluidine blue. Plants were inoculated with the *Rhizobium leguminosarum* bv. *viciae* 3841 strain. Scale bars: 500 µm (**A**), 40 µm (**B**,**D**,**F**), and 1 μm (**C**,**E**,**G**).

**Figure 3:**
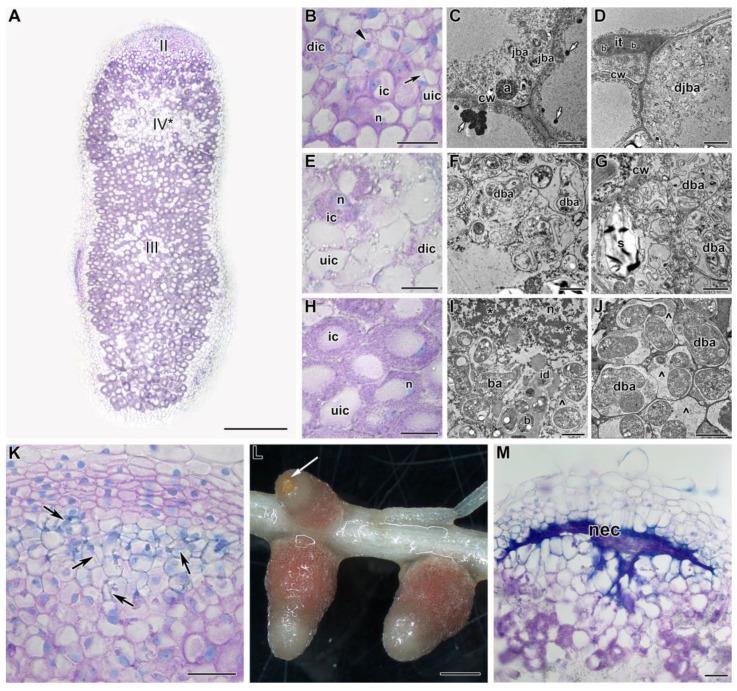
Histological and ultrastructural organization in nodules of the pea (*Pisum sativum*) line SGE in 5 days of exposure to 28 °C. (**A**) General view. (**B**,**E**,**H**,**K**,**M**) Histological and (**C**,**D**,**F**,**G**,**I**,**J**) ultrastructural organization of nodule tissue. (**L**) Nodule phenotype. Cells from the infection (**B**–**D**), apical senescence (**E**–**G**), and nitrogen fixation (**H**–**J**) zones. (**K**) Infection thread network clearly seen in some nodules with degraded cells in the infection zone. (**L**) An example of a nodule with a brown spot on the nodule top. (**M**) Necrotic cells in the place of the infection zone. II, infection zone; III, nitrogen fixation zone; IV*, senescence zone at the apical part of the nodule. ic, infected cell; uic, uninfected cell; dic, degraded infected cell; n, nucleus; it, infection thread; id, infection droplet; cw, cell wall; b, bacterium; ba, bacteroid; jba, juvenile bacteroid; djba, degraded juvenile bacteroid; dba, degraded bacteroid; a, amyloplast; s, starch; *, coarse chromatin; ^, multibacteroid symbiosome; nec, necrotic cells. Black arrows indicate infection threads, the triangle indicates an infection droplet, small white arrows indicate electron-dense inclusions in the vacuole, and a large white arrow indicates a brown spot. Histological sections were stained with toluidine blue. Plants were inoculated with the *Rhizobium leguminosarum* bv. *viciae* 3841 strain. Scale bars: 1 mm (**L**), 500 µm (**A**), 50 µm (**K**,**M**), 40 µm (**B**,**E**,**H**), 5 μm (**C**,**D**), and 1 μm (**F**,**G**,**I**,**J**).

**Figure 4:**
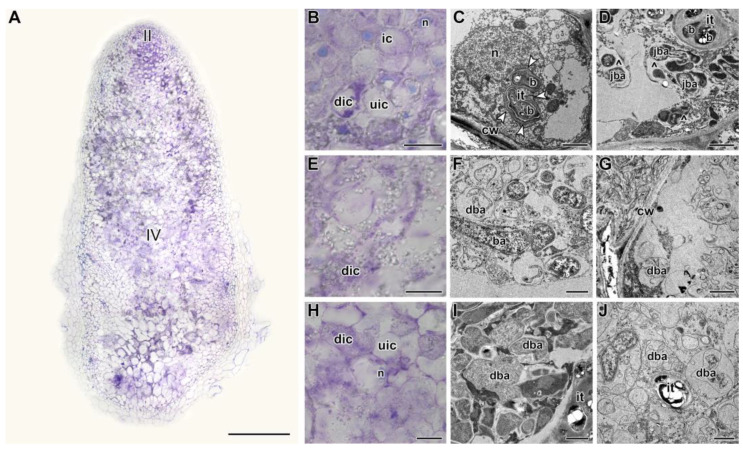
Histological and ultrastructural organization in nodules of the pea (*Pisum sativum*) line SGE in 9 days of exposure to 28 °C. (**A**) General view. (**B**,**E**,**H**) Histological and (**C**,**D**,**F**,**G**,**I**,**J**) ultrastructural organization of nodule tissue: cells from the infection (**B**–**D**), apical senescence (**E**–**G**), and senescence (**H**–**J**) zones. II, infection zone; IV, senescence zone. ic, infected cell; uic, uninfected cell; dic, degraded infected cell; n, nucleus; it, infection thread; b, bacterium; ba, bacteroid; jba, juvenile bacteroid; dba, degraded bacteroid; cw, cell wall; ^, multibacteroid symbiosome. White arrowheads indicate outgrowths of the infection thread wall. Histological sections were stained with toluidine blue. Plants were inoculated with the *Rhizobium leguminosarum* bv. *viciae* 3841 strain. Scale bars: 500 µm (**A**), 40 µm (**B**,**E**,**H**), 5 μm (**C**), and 1 μm (**D**,**F**,**G**,**I**,**J**).

Nodules were analyzed after exposure of plants to 21 °C or 28 °C for 1, 5, and 9 days (Figure 2, Figure 3 and Figure 4 and Figures S1 and S2). After 1 day of plant exposure, no differences in nodule histological organization were revealed (Figure 2A,B,D,F and Figure S1A,B,D,F). However, at the ultrastructural level, some abnormalities were observed in heat-stressed nodules (Figure 2C,E,G). In the infection zone, some bacteria released from infection droplets underwent degradation (Figure 2C). In the nitrogen fixation zone, the peribacteroid space was enlarged, and bacteroids contained electron-dense inclusions (Figure 2E) and poly-β-hydroxybutyrate (PHB) granules (Figure 2G). In the heat-unstressed nodules, the usual ultrastructural organization for indeterminate nodules was observed (Figure S1C,E,G).

In nodules heat-stressed for 5 days, in the infection zone, some cells were degraded, as was clearly seen at both histological (Figure 3A,B) and ultrastructural (Figure 3D) levels. In vacuoles, osmiophilic material, was accumulated (Figure 3C). In some nodules, cells in the infection zone underwent profound degradation, resulting in a clearly visible network of infection threads (Figure 3K). However, the most striking change was associated with active cell degradation in the distal part of the nitrogen fixation zone and the formation of a senescence zone in the apical part of the nodule (Figure 3A,E). The degraded cells were filled with symbiosomes containing degraded (Figure 3F) and ‘ghosts’ of bacteroids (Figure 3G). At the initial stages of degradation in infected cells, the formation of coarse clumps of chromatin and multibacteroid symbiosomes, as well as fragmentation and vacuolization of the rough endoplasmic reticulum (ER), (Figure 3I) was observed. A significant accumulation of amyloplasts was noted (Figure 3E,G). At the base of the nodule, some degraded cells were also presented (Figure 3J). It should be noted that starting from this period of exposure to elevated temperature, a brown spot was noticeable on the tops of some nodules (Figure 3L). This spot was formed by the cells of the infection zone that had undergone necrotic changes (Figure 3M).

In nodules heat-stressed for 9 days, degradation covered the cells of almost the entire nodule (Figure 4A). In the infection zone, many cells were degraded (Figure 4B), walls of infection threads were thickened, stratified, and formed various outgrowths, rough ER was fragmented and vacuolized (Figure 4C), multibacteroid symbiosomes were formed with degraded bacteroids (Figure 4D). Both in the distal part (Figure 4F,G) and at the base of the nodule (Figure 4I,J), infected cells were observed degrading to varying degrees. At the same time, the histological (Figure S2A,B,D) and ultrastructural organization (Figure S2C,E) were preserved in the heat-unstressed nodules of the same age. At the base of the nodule, the formation of the senescence zone was initiated (Figure S2A,F,G).

### 2.3. Expression Analysis of the Genes Associated with Senescence, Heat, and Defense Responses in Heat-Stressed and Heat-Unstressed Nodules in the Line SGE

**Figure 5:**
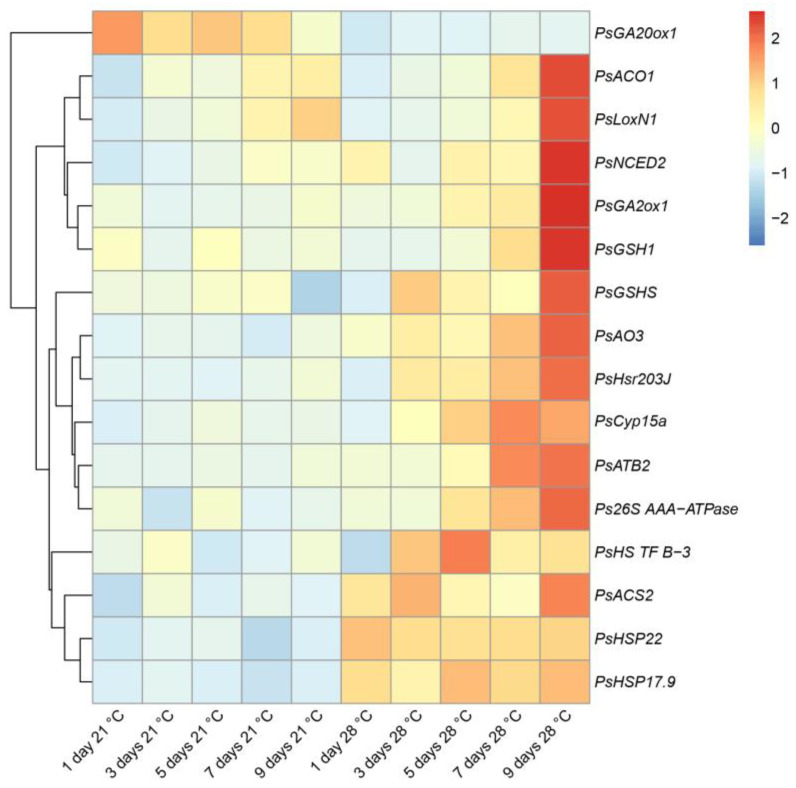
Heatmap showing relative gene expression levels in heat-unstressed and heat-stressed nodules of the pea (*Pisum sativum*) line SGE in 1, 3, 5, 7, and 9 days of exposure. Transcript levels were determined by real-time PCR and calculated using the ΔCt method with glyceraldehyde-3-phosphate dehydrogenase (*PsGapC1*) serving as the reference gene. The color scale shows relative expression values for each gene after Z-transformation. Gene expression levels were compared using one-way ANOVA; a *p*-value < 0.05 was considered significant. Plants were inoculated with the *Rhizobium leguminosarum* bv. *viciae* 3841 strain.

To characterize heat stress-induced changes in gene expression, we analyzed marker genes for which increased expression was previously shown to be enhanced during symbiotic nodule senescence [34,35] and the development of defense responses [36,37]. In this study, this set of genes was supplemented with heat-shock protein genes. Most of the analyzed genes showed an increase in expression levels after 9 days of exposure to elevated temperature compared to those after 1 day of exposure (Figure 5). The maximum level of expression of the majority of analyzed genes after 9 days of exposure to elevated temperature coincides with the most pronounced degradation of symbiotic structures observed in this period of analysis (Figure 4). However, for the *PsHSP22* gene, the activation of the maximum expression level was observed after 1 day of exposure, and for the *PsHS TF B-3* gene, in 5 days (Figure 5). The *PsHSP22*, *PsHSP17.9*, *PsACS2*, and *PsNCED2* genes were characterized by an increase in expression levels in heat-stressed nodules already in 1 day of elevated temperature exposure compared to the heat-unstressed nodules (Figure 5).

At the same time, the expression level of the gene encoding the gibberellic acid (GA) biosynthesis enzyme, GA-20 oxidase 1 (*PsGA20ox1*), was significantly suppressed in the heat-stressed nodules compared to the heat-unstressed nodules during the entire exposure period (Figure 5).

### 2.4. The Transcriptomic Analysis of Heat-Stressed and Heat-Unstressed Nodules in the Line SGE

**Figure 6:**
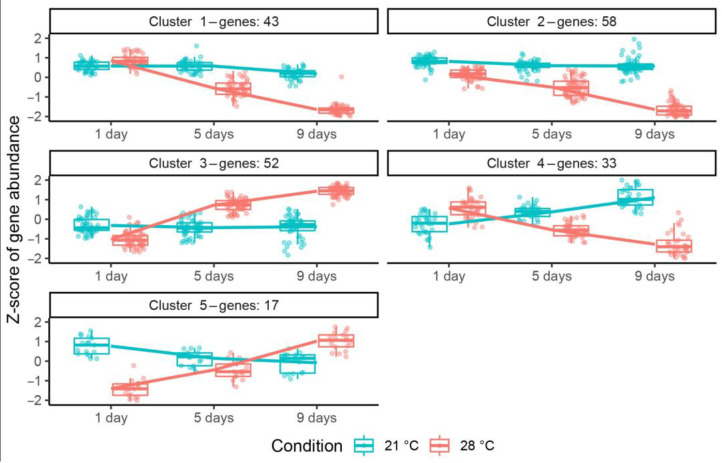
Clustering of differentially expressed genes in heat-unstressed and heat-stressed nodules of the pea (*Pisum sativum*) line SGE in 1, 5, and 9 days of exposure. Temperature conditions are color coded: 21 °C in blue, 28 °C in red. Plants were inoculated with the *Rhizobium leguminosarum* bv. *viciae* 3841 strain.

The transcriptomic data presented in this study are openly available at NCBI SRA under the accession number PRJNA991148. Analysis revealed, in total, 258 genes differentially expressed during exposure to elevated temperature (Figure S3): the expression of 161 genes was decreased (Table S2), and for 97 genes it was increased (Table S2). The cluster analysis subdivided differentially expressed genes (DEGs) with common expression changes into five clusters (Figure 6). Genes from clusters 1 (43 genes) and 2 (58 genes) showed a drastic decrease in gene expression in heat-stressed nodules, while their expression was slightly decreased or not changed in heat-unstressed nodules. Cluster 3 was presented by 52 genes significantly upregulated in heat-stressed nodules, whose expression was slightly altered or not changed in heat-unstressed nodules. Genes from cluster 4 (33 genes) were characterized by their downregulation in heat-stressed nodules and upregulation in heat-unstressed nodules, while cluster 5 (17 genes) showed an opposite pattern.

Gene Ontology enrichment analysis (regarding biological process GO terms) of DEGs from these clusters showed that in heat-stressed nodules, downregulated genes (clusters 1, 2, 4) shared no GO terms with upregulated ones (clusters 3, 5). Significantly enriched GO terms for clusters with downregulated genes included nodule morphogenesis, defense response, response to reactive oxygen species, response to stress, cellular response to phosphate starvation, and the thiamine biosynthetic process. Genes from cluster 3 were characterized as related to metabolic processes, sulfate assimilation, and the sulfur amino acid metabolic process, while cluster 5 genes were related to the catabolism of polysaccharides.

Several genes showed no or little changes in expression in heat-unstressed nodules throughout the experiment and in heat-stressed nodules for 1 day but were significantly downregulated after 5 and 9 days of exposure to 28 °C, while some genes demonstrated a similar pattern with slight variations (Figure S4). Another group of genes showed a sharp decrease in expression, specifically after 9 days of exposure to elevated temperature but not after 1 or 5 days, with no or little expression changes in unstressed nodules (Figure S5). Genes from cluster 3 with several other genes were moderately upregulated or unchanged in heat-unstressed nodules but showed upregulation in heat-stressed nodules, especially in 5 days compared to 1 day of exposure to elevated temperature. Other genes demonstrated a similar pattern but with downregulation in heat-unstressed nodules in 9 days (Figure S6). Finally, some genes were downregulated in nodules exposed to 28 °C for 1 day compared to heat-unstressed nodules but restored their expression or became upregulated in 9 days of exposure (Figure S7).

### 2.5. Analysis of Nodule Recovery in Plants of Line SGE Transferred from Elevated Temperature (28 °C) to Optimal One (21 °C)

**Figure 7:**
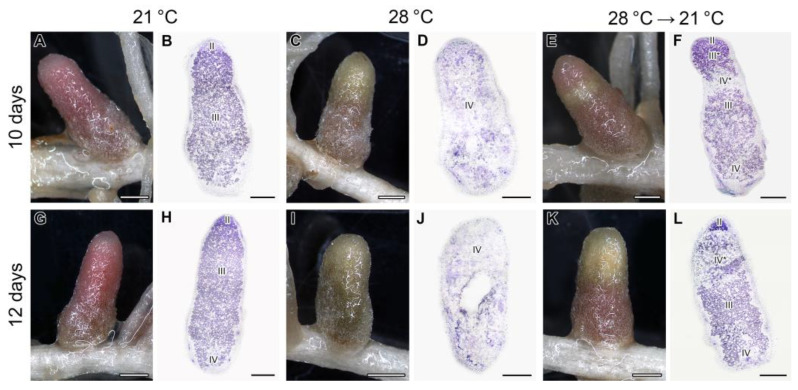
Nodules (**A**,**C**,**E**,**G**,**I**,**K**) and their histological organization (**B**,**D**,**F**,**H**,**J**,**L**) in the pea (*Pisum sativum*) plants of the line SGE exposed to 21 °C (**A**,**B**,**G**,**H**), exposed to 28 °C (**C**,**D**,**I**,**J**), and transferred back from 28 °C to 21 °C (**E**,**F**,**K**,**L**). Nodules were analyzed 10 days after the beginning of the experiment (**A**–**F**) or in 12 days (**G**–**L**). II, infection zone; III, nitrogen fixation zone; III*, recovered nitrogen fixation zone; IV, senescence zone; IV*, senescence zone in the apical part of the nodule. Sections were stained with toluidine blue (**B**,**D**,**F**,**H**,**J**,**L**). Plants were inoculated with the *Rhizobium leguminosarum* bv. *viciae* 3841 strain. Scale bars: 1 mm (**A**,**C**,**E**,**G**,**I**,**K**) and 500 µm (**B**,**D**,**F**,**H**,**J**,**L**).

The nodules exposed to elevated temperature for 3 and 5 days manifested the same phenotypes as previously described (Figure 1A–D). Nodules exposed to 21 °C for 10 days showed pink color and typical histological organization (Figure 7A,B). In contrast, heat-stressed nodules for 10 days demonstrated green and brown color, and the senescence zone occupied the whole nodule (Figure 7C,D). In many nodules transferred after 3 days of elevated temperature exposure to optimal temperature, a green strip close to the apical part of the nodule was clearly seen (Figure 7E). However, above this strip, a pink zone was observed (Figure 7E). The green strip was presented by degraded cells forming a senescence zone in the apical part of the nodule, while the pink zone was likely presented by a newly formed nitrogen fixation zone (Figure 7F).

**Figure 8:**
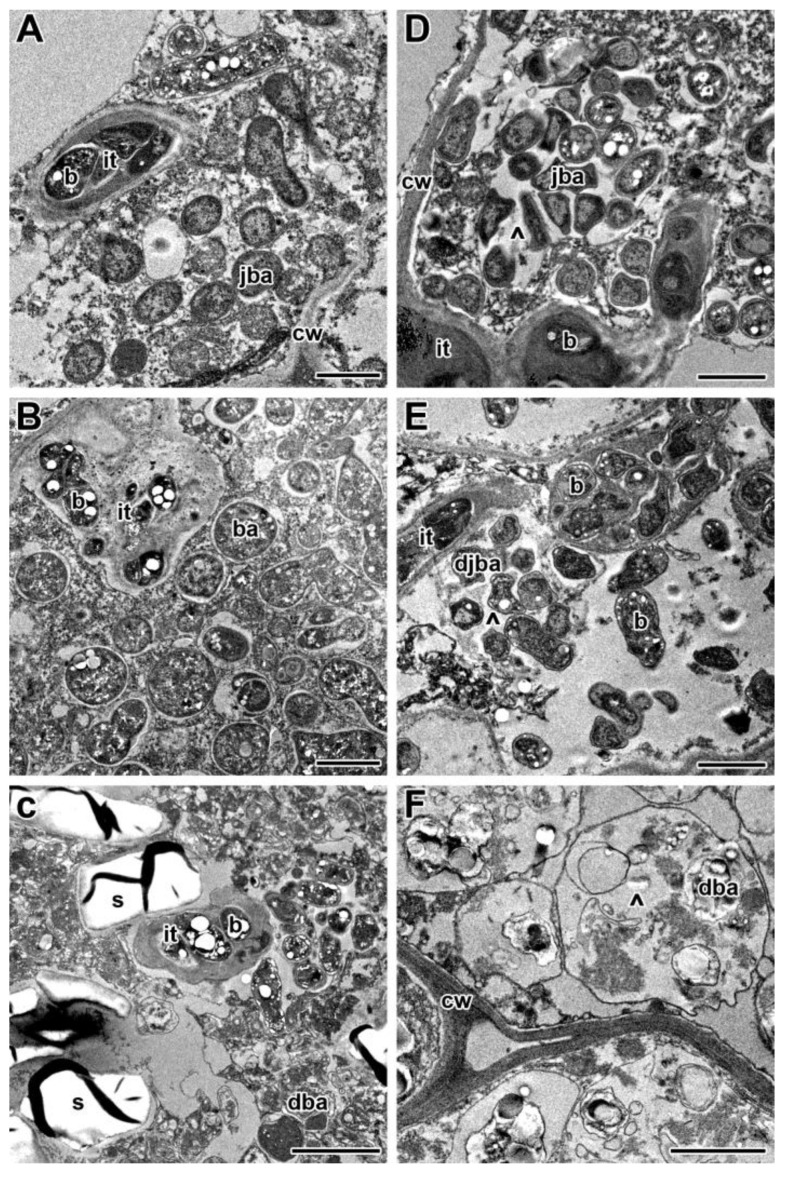
Ultrastructural organization in nodules of pea (*Pisum sativum*) SGE plants transferred back to optimal temperature (21 °C) after exposure to 28 °C for 3 (**A**–**C**) and 5 (**D**–**F**) days. Cells from the recovered infection zone (**A**,**D**), newly formed nitrogen fixation zone (**B**), and senescence zone in the nodule center (**C**–**F**). it, infection thread; b, bacterium; ba, bacteroid; jba, juvenile bacteroid; djba, degraded juvenile bacteroid; dba, degraded bacteroid; cw, cell wall; s, starch; ^, multibacteroid symbiosome. Scale bars: 1 μm.

The formation of the newly formed nitrogen fixation zone demonstrated at least partial recovery of nodules after their transfer to optimal temperature after three days of heat stress. The detailed analysis of nodule ultrastructural organization revealed the normal structure of the infected cells in the infection zone (Figure 8A) and in the newly formed nitrogen fixation zone (Figure 8B). Some bacteroids in infected cells in the latter zone showed an unusual spherical shape and accumulation of PHB granules (Figure 8B). In the centrally located senescence zone, infected cells with degraded bacteroids and numerous starch granules were located (Figure 8C).

**Figure 9:**
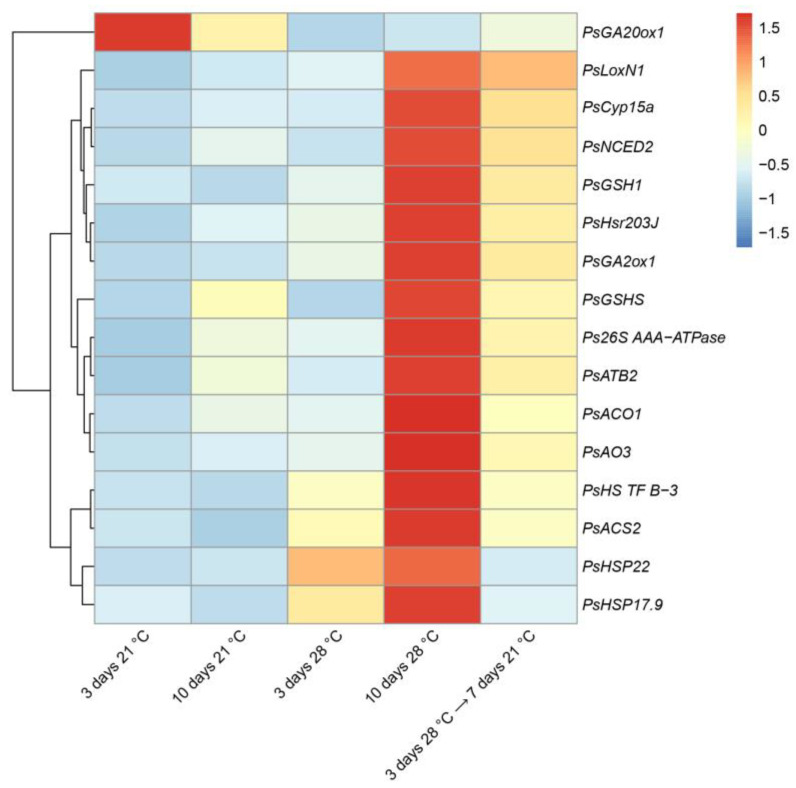
Heatmap showing relative gene expression levels in nodules of pea (*Pisum sativum*) plants of the line SGE exposed to 21 °C, exposed to 28 °C, and transferred back from 28 °C to 21 °C. Transcript levels were determined by real-time PCR and calculated using the ΔCt method with glyceraldehyde-3-phosphate dehydrogenase (*PsGapC1*) serving as the reference gene. The color scale shows relative expression values for each gene after Z-transformation. Gene expression levels were compared using one-way ANOVA; a *p*-value < 0.05 was considered significant. Plants were inoculated with the *Rhizobium leguminosarum* bv. *viciae* 3841 strain.

The recovery was also confirmed at the transcriptional level. The levels of almost all analyzed genes were significantly lower in the nodule transferred after 3 days of elevated temperature exposure to optimal temperature than in nodules exposed to heat stress for 10 days (Figure 9). In particular, levels of expression were decreased for genes of heat-shock proteins (*PsHSP17.9*, *PsHSP22*).

In nodules exposed to 21 °C for 12 days, a green color was seen at the base of the nodule, which indicated the formation of the senescence zone (Figure 7G,H). The nodules heat-stressed for 12 days were green and brown, and at the histological level, a deep degradation was observed, including the formation of a cavity in some nodules (Figure 7I,J). In nodules transferred after 5 days of elevated temperature exposure to optimal temperature, a recovery of nitrogen-fixing cells was not observed (Figure 7K,L). In the infection zone, after the release of bacteria from infection droplets, multibacteroid symbiosomes were formed, in which juvenile bacteroids were subjected to degradation (Figure 8D). Further signs of degradation intensified (Figure 8E). As a result, degraded infected cells were filled with ‘ghosts’ of bacteroids (Figure 8F).

### 2.6. Exposure of Plants of the Line SGE Inoculated with R. leguminosarum *bv.* viciae 3841 for Some Time to Optimal Temperature Leveled the Negative Effect of Elevated Temperature

Exposure of plants of the line SGE inoculated with *R. leguminosarum* bv. *viciae* 3841 for 7 h to optimal temperature (21 °C) with previous and subsequent gradient decrease and increase in temperature from/to 28 °C for 1.5 h and exposure of plants to elevated temperature for 14 h did not cause pronounced changes in nodule morphology (Figure S8).

## 3. Discussion

In this study, the effect of elevated temperature on the functioning of nodules formed by *R. leguminosarum* bv. *viciae* 3841 strain on the pea line SGE was investigated. This symbiotic pair is actively used as a model system for various studies of nodule development in peas [32,37,38,39,40]. Heat-stressed nodules changed their color from pink to green at their apical parts (Figure 1), which can be considered as the induction of degradation associated with leghemoglobin oxidation [41,42]. In indeterminate nodules, natural and induced senescence is always activated in the basal part of the nodule [23,25,31,33,35,43]. The only study that examined the effect of temperature stress (30 °C) on the structure of *T. subterraneum* nodules showed that senescence was induced in the basal part of the nodule [15]. Thus, our study discovered the possibility of the formation of a senescence zone in an anomalous place in the apical part of the nodule.

Detailed analyses of the histological and ultrastructural organization of SGE line nodules, as well as the expression of marker genes, confirmed the formation of the senescence zone in the apical part of the nodule, affecting the infection zone and the distal part of the nitrogen fixation zone (Figure 2, Figure 3, Figure 4 and Figure 5). Indeed, at the early stages of degradation, the formation of multibacteroid symbiosomes was observed (Figure 3I), and coarse clumps of chromatin formed in the nuclei (Figure 3I), which can lead to inactivation of active transcription sites, considered a precursor of cell death [44,45]. Subsequently, degradation of bacteroids was observed in symbiosomes (Figure 3F), resulting in the formation of ‘ghosts’ of bacteroids (Figure 3G). In peas, multibacteroid formation is a common feature for ineffective symbiosis as a result of unfavorable conditions, like cadmium stress [31,32] and fungicide treatment [39,40]. Mutations in some pea symbiotic genes (*PsSym31*, *PsSym32*, *PsSym40*, *PsSym33*) also lead to the formation of multibacteroid symbiosomes [46,47,48,49]. In *Medicago truncatula* Gaertn., *rsd* (regulator of symbiosome differentiation) mutants also form multibacteroid symbiosomes [50]. Although the ER is a highly dynamic structure changing in response to various morphogenetic stimuli, the fragmentation and vacuolization of the ER observed in infected and colonized nodule cells in heat-stressed plants negatively affect protein synthesis and processing in these cells. Similar changes in ER were observed in infected nodule cells of *Medicago sativa* L. under nitrate treatment [51] and in pea nodules of the ineffective mutant *sym40–1* [52]. It is worth noting the downregulation of genes encoding components of ER in heat-stressed nodules for 5 days (Figure S4). Notably, in 3-week-old nodules exposed to elevated temperature for 12 days, cavities were formed within the nodules (Figure 7J). Previously, the formation of such cavities was reported only for 6-week-old pea nodules [53].

At the transcriptional level, genes associated with senescence, heat, and defense responses in heat-stressed nodules were activated in the line SGE. Among the genes associated with senescence were genes of proteases (*PsCyp15a* and *Ps26S AAA-ATPase*) (Figure 5). Previously, the cysteine protease genes were shown to be the most strongly and specifically activated genes during the natural and induced senescence of nodules of various legumes [23,28,33,34,35,43,54,55,56,57,58]. An increase in the transcript abundance of the components of the 26S proteasome pathway was shown during the aging of *M. truncatula* nodules [33,43]. One more gene associated with senescence was the *PsATB2* gene encoding the bZIP transcription factor (Figure 5). An increase in the transcript abundance of the *ATB2* and other members of the bZIP family was shown during natural and induced senescence of *P. sativum*, *M. truncatula*, and *G. max* symbiotic nodules [33,34,35,59,60,61]. In *M. truncatula* and *P. sativum*, it was shown that ethylene, abscisic acid, and jasmonic acid contributed to the natural and induced senescence of the nodule, while gibberellins had a negative effect on nodule aging [34,43,53,58]. This study demonstrated a downregulation of the transcript abundance of the GA biosynthesis gene, *PsGA20ox1,* and an upregulation of the transcript level of the GA deactivation gene, *PsGA2ox1,* in heat-stressed nodules (Figure 5), which coincides with the negative effect of gibberellins on nodule senescence. The positive effect of ethylene, abscisic acid, and jasmonic acid on nodule senescence is supported in this study by the activation of genes for biosynthesis of ethylene (*PsACS2*, *PsACO1*), jasmonic acid (*PsLoxN1*), and abscisic acid (*PsNCED2*, *PsAO3*) (Figure 5).

In this study, an upregulation of gene expression for heat-shock proteins (*PsHSP17.9*, *PsHSP22*) and heat stress transcription factor (*PsHS TF B-3*) in heat-stressed nodules was shown. Previously, upregulation of *HSP* and *HSF* orthologues was reported during the natural senescence of *Lotus japonicus* (Regel) K.Larsen and *M*. *truncatula* nodules [43,56].

An increase in the expression level of the hypersensitivity response marker gene, *PsHsr203J*, was detected in heat-stressed nodules, which may indicate the activation of defense reactions. Previously, a high level of *PsHsr203J* transcripts was found in nodules of *P. sativum* mutant lines characterized by premature degradation of symbiotic structures, including the *sym40–1* mutant characterized by oxidative stress [36]. In tobacco, *Hsr203* encodes a serine hydrolase with esterase activity (scavenger of reactive oxygen species) [62], which can indicate its involvement in oxidative stress response. The upregulation of genes encoding the GSH biosynthesis enzymes (*PsGSH1*, *PsGSHS*) in heat-stressed nodules observed in this study may also indicate the link to oxidative stress. Indeed, in early senescent nodules of the mutant *sym40–1*, the genes of glutathione biosynthesis were upregulated, while the content of GSH was low compared to the wild type [37].

Transcriptome analysis was used for a more detailed study of transcriptional activity in heat-stressed nodules (Table S2). The early expression activation of the heat-shock proteins *HSP70*, *HSP90*, and *HSP22* genes in heat-stressed nodules for 1 day (Figure S4) coincides with the first signs of nodule degradation observed in these nodules (Figure 2).

Unsurprisingly, several stress-related genes, such as for dehydrin and dirigent-like proteins, which are known agents of plant tolerance to various abiotic stresses [63,64], were upregulated. Similarly, there was an upregulation of genes from families involved in plant response to various environmental stresses: the A20-type zinc finger Psat5g256720 gene [65] and the PHOS32 universal stress protein Psat6g162080. Interestingly, the homolog of the latter gene in *M. sativa* (Medtr1g087200) was reported to be upregulated in the nematode-resistant cultivar [66], and similar genes for PHOS32-like proteins were upregulated in response to the *Fusarium* infection in pea [67].

The pathogen resistance-related genes (gamma-thionin family, cysteine-rich secretory protein, chitinases, and xylanase inhibitors) demonstrated different patterns during exposure to 28 °C (Figures S3–S6), possibly because pathogen resistance in plants can be differently regulated by temperature stress [68,69,70]. Moreover, the *PsHsr203J* hypersensitive response gene was also upregulated in heat-stressed nodules for 5 and 9 days, as has already been demonstrated using real-time PCR analysis (Figure 5). Similar upregulation of the genes for aspartyl and cysteine proteases, as well as the gene for cysteine protease inhibitor, most likely reflects temperature-induced senescence in heat-stressed nodules. Certain cysteine protease inhibitors, or cystatins, were shown to be upregulated during nodule senescence in soybean [71], while others were proposed to be involved in the resistance to abiotic stresses [72]. Upregulation was also observed for senescence-related genes (*SRG1*-like and *YLS9*-like). These findings confirm active senescence in heat-stressed nodules for 5 days (Figure 3).

The appearance of a senescence zone in the distal part of the nitrogen fixation zone was followed by gene downregulation in heat-stressed nodules in 5 days (Figure S4). For instance, one of the most downregulated genes was the α-carbonic anhydrase gene. These enzymes are involved in nodule development and functioning [73,74]. A similar pattern was found for the gene encoding nicotianamine synthase protein, the homologs of which in *M. truncatula* were reported to be expressed in nodules, controlling iron trafficking crucial for nitrogenase functioning [75]. Other downregulated genes for enzymes involved in carbon assimilation have also been shown to be associated with nodule metabolism [76]. The effect of the elevated temperature on nitrogen fixation was also manifested in the downregulation of genes for enzymes crucial for the assimilation and transport of fixated nitrogen (glutamine synthetase, asparagine synthase) in amide-transporting legumes [77] such as pea, as well as downregulation of several leghemoglobin genes. A decrease in leghemoglobin levels accompanies both nodule senescence [43] and senescence induced by various abiotic factors such as salt [78,79], water and nitrate stress [80], drought [20], or darkness [24,25]. Some of these genes were slightly suppressed even in heat-stressed nodules for only one day, although no visible morphological changes were revealed. At the same time, an upregulation of the asparaginase gene was observed (Figure S6). Asparaginase, which catalyzes the hydrolysis of asparagine, was shown to be inactivated in developed *Lupinus angustifolius* L. nodules [81,82]. According to transcriptomic data on infected cells from different zones of pea nodules, the asparaginase gene demonstrated 14-fold downregulation in the nitrogen-fixing cells compared to the cells from the infection zone [38].

The group of downregulated genes in heat-stressed nodules for 5 days included beta-tubulin and expansin genes (Figure S4). Previously, it was shown that rearrangements of tubulin cytoskeleton were involved in bacteria accommodation and symbiosome arrangement in pea nodules [83], and expansins were associated with the growth of infected cells [84,85]. According to the data on transcriptomic changes in the infected pea nodule cell, the maximum expression of these genes was associated with the nitrogen fixation zone [38].

After 9 days in heat-stressed nodules, there was a decline in the expression of some genes involved in the development of the infection thread, such as genes for pectin acetyl esterase and proline-rich extensin (Figure S5). This may indicate the cessation of the infection process, which usually persists for a long time in the nodules. Moreover, during the natural senescence of the nodule, infection threads are preserved, and bacteria emerge from them, passing to a saprophytic lifestyle [86]. Interestingly, the gene for DEMETER-like protein was also downregulated in the same manner. Proteins from this family are involved in the demethylation of DNA and were shown to control the late stages of nodule development in *M. truncatula* [87,88]. Late and early nodulin genes (including those coding NCR- and NGR-peptides) were also downregulated. However, the expression of the Psat6g034320 gene coding protein with a signal peptide peptidase signature, the homolog of which in *M. truncatula* (Medtr1g008280) was proposed as NCR-peptidase [89], decreased already after 5 days of exposure to 28 °C.

In this study, the possibility of recovery of heat-stressed nodules was checked. It was shown that after 3 days of heat stress, at least partial recovery of the nodule phenotype at both transcriptional and cellular levels was possible, but not after 5 days (Figure 7, Figure 8 and Figure 9). Previously, in *T. subterraneum* plants transferred back from high temperature (30 °C) to the optimum temperature (22 °C), nitrogenase activity was restored, and the level of activity was strain dependent [15]. The exposure time (5 days) identified in this study as critical for the occurrence of irreversible damage to the nodule coincides with the minimum duration of the heat wave [90].

Exposure of plants to elevated temperatures for 24 h in temperate latitudes is unlikely; obviously, temperatures can decrease at night. When growing pea plants of the SGE line during the day with a gradual decrease in temperature at night, the nodules formed with *R. leguminosarum* bv. *viciae* 3841 did not show visible signs of senescence induction (Figure S8).

## 4. Materials and Methods

### 4.1. Plant Material, Bacterial Strains, and Plant Growth Conditions

The pea (*Pisum sativum* L.) laboratory line SGE [91] from the Collection of Pea Genotypes (All-Russia Research Institute for Agricultural Microbiology) was used in this study. Protocol for seed sterilization was described earlier [36]. Seedlings were inoculated with the *Rhizobium leguminosarum* bv. *viciae* 3841 strain from the Russian Collection of Agricultural Microorganisms (All-Russia Research Institute for Agricultural Microbiology) [92]. Plants were grown in a growth chamber MLR-352H (Sanyo Electric Co., Ltd., Moriguchi, Japan) under controlled conditions: day/night, 16/8 h; temperature, 21 °C; relative humidity 75%; photosynthetic photon flux density of ~280 μmol photons m^−2^ s^−1^ for 3 weeks after inoculation (WAI), with the following change in growth conditions in accordance with the design of the experiment.

### 4.2. Growing Pea Plants at Elevated Temperature

After 3 WAI, half of the planted plants were transferred to another growth chamber under the same growth conditions, only instead of 21 °C, an elevated temperature of 28 °C was maintained. The other half of the plants were used as a control. The material for analysis was harvested in 1, 3, 5, 7, and 9 days after the beginning of exposure to elevated temperature (Figure S9).

To study the recovery of nodules exposed to elevated temperature, pea plants of the line SGE, after growing at 28 °C for 3 and 5 days, were transferred back to the first growth chamber and grown at 21 °C for 7 days (10 and 12 days of exposure, respectively), followed by the harvest of material for further analysis. Nodules of plants grown for 3, 5, 10, and 12 days at 21 °C or 28 °C from the start of exposure were used as controls (Figure S10).

Finally, the effects of gradient temperature changes on pea nodules were studied by gradually changing temperature: exposure to 28 °C for 14 h followed by a gradient decrease to 21 °C for 1.5 h, exposure to 21 °C for 7 h followed by a gradient increase to 28 °C for 1.5 h.

### 4.3. Light Microscopy Analysis

Plant roots with symbiotic nodules were photographed using a SteREO Lumar V12 stereomicroscope (Carl Zeiss, Oberkochen, Germany) equipped with a camera AxioCam MRc 5 (Carl Zeiss).

Nodules of the line SGE exposed to elevated temperature for 1, 5, and 9 days, as well as nodules from plants after recovery and appropriate controls, were fixed in 3% paraformaldehyde in MTSB buffer (50 mM PIPES, 5 mM MgSO_4_·7H_2_O, 5 mM EGTA, pH 6.9) and then rinsed, dehydrated, and embedded in Steedman’s wax as described earlier [34]. Longitudinal serial sections 10 µm thick were obtained using an HM-360 microtome (Microm International GmbH, Walldorf, Germany). Sections were mounted on glass slides and de-waxed by incubation in 96% ethanol at 28 °C overnight. Then, sections were rehydrated through a series of ethanol/water solutions and placed in TBS buffer [34]. Sections were stained with toluidine blue (0.05% solution in TBS) and mounted in TBS.

Light microscopy analysis of nodule sections of heat-unstressed and exposed to 28 °C plants was performed using AxioImagerZ1 (Carl Zeiss), equipped with an Axiocam 506 color camera (Carl Zeiss). Images were analyzed using the ZEN 2 core SP1 software version 2.0 (Carl Zeiss).

### 4.4. Electron Microscopy Analysis

Nodules of the line SGE exposed to elevated temperature for 1, 5, and 9 days, as well as nodules from plants after recovery, were harvested from roots and placed directly in fixative. The whole nodules with glancing cut on one side were fixed in 2.5% glutaraldehyde (Sigma-Aldrich, St. Louis, MO, USA) in 0.1 M phosphate buffer, pH 7.2, and post-fixed in 2% osmium tetroxide in phosphate buffer for 2 h. The samples were subjected to sample preparation as described earlier [35]. Dehydration and progressively embedding with Eponate 12 (Ted Pella, Inc., Redding, CA, USA) was carried out using an Automated Tissue Processor Leica EM TP (Leica Microsystems, Vienna, Austria). Embedded samples were transferred to blocks in fresh resin and polymerized at 60 °C for 48 h in a Memmert IN55 incubator (Memmert GmbH, Schwabach, Germany).

For transmission electron microscopy, 90–100 nm thick ultrathin sections were obtained on a Leica EM UC7 ultramicrotome (Leica Microsystems) and collected on copper grids coated with formvar and carbon. The grids containing the sections were counterstained with 2% aqueous uranyl acetate for 1 h, followed by lead citrate for 1 min. The nodule tissues were examined and photographed under a Tecnai G2 Spirit electron microscope (FEI, Eindhoven, The Netherlands) at 80 kV. Digital micrographs were taken with a MegaView G2 CCD camera (Olympus-SIS, Münster, Germany).

### 4.5. Extraction of Total RNA and cDNA Synthesis

Total RNA was extracted from nodules ground in liquid nitrogen using the RNeasy Plant Mini Kit (Qiagen, Hilden, Germany) following the manufacturer’s instructions. Total RNA was quantified with a Qubit 2.0 Fluorimeter (Invitrogen, Carlsbad, CA, USA) according to the manufacturer’s protocol. RevertAid Reverse Transcriptase (MBI Fermentas, Vilnius, Lithuania) was used to synthesize cDNA from 2.5 μg of total RNA treated with DNase I (MBI Fermentas) under the conditions recommended by the manufacturer.

### 4.6. Relative Real-Time PCR Analysis

The primer design was performed using the VectorNTI Advanced 10 software version 11.5.1 (Invitrogen). Primers (Table S1) [34,36,37,53,93,94,95] were synthesized by Evrogen (Moscow, Russia). A relative real-time PCR analysis was performed as described earlier [34]. The relative expression level was calculated with the ΔCt method. The *PsGapC1* gene (L07500.1, Table S1) was used as a reference gene. Statistical treatment of experimental results was processed using Microsoft Excel 2016. The experiments were carried out in three biological replicates with five to six plants per variant. Relative expression levels were visualized using the pheatmap package version 1.0.12 [96].

### 4.7. Transcriptomic Analysis

The 3′ MACE sequencing libraries were prepared from RNA samples using a 3′ MACE kit (GenXPro GmbH, Frankfurt am Main, Germany) and sequenced on Illumina HiSeq X Ten at Macrogen (Seoul, Republic of Korea). DEGs were identified using the DESeq2 package version 1.28.1 [97] with the likelihood-ratio test (adjusted *p*-value < 0.05) using the full model X ~ Condition+Time+Condition:Time and reduced model without the interaction term. Clustering of genes was performed with the DEGreport package version 1.24.1 [98]; the DESeq2 and ggplot2 version 3.3.6 [99] packages were used to visualize gene expression profiles. GO enrichment analysis for each cluster was carried out with the topGO package version 2.44.0 [100].

## 5. Conclusions

Under inoculation with *R. leguminosarum* bv. *viciae* 3841, elevated temperature induced an unusual senescence pattern in the apical rather than basal part of the nodule. At the morphological and transcriptional levels, the observed abnormal senescence was similar to the previously described senescence, which is always induced in the basal part by other stressors. Therefore, further studies should be aimed at deciphering the molecular mechanisms leading to senescence in the apical part of pea nodules under heat stress, with the involvement of other omics technologies. It has been shown that nodule recovery is possible after exposure to elevated temperature for 3 days but not after 5 days. It is noteworthy that the duration of the heat wave is at least 5 days. This observation indicates that nitrogen fixation can often cease completely in legumes during the onset of heat waves, which can lead to nitrogen deficiency and subsequent yield loss. It seems that it could be important to analyze various pea genotypes and *R. leguminosarum* strains in order to find those plant–microbe combinations resistant to heat stress. These combinations will be a useful tool for pea breeders to select new varieties adapted to global warming.

## Data Availability

The data presented in this study are openly available at NCBI SRA under the accession number PRJNA991148.

## Supplementary Materials

The following supporting information can be downloaded at: https://www.mdpi.com/article/10.3390/ijms242417144/s1.
