# Supplementary material for: Effects of Elevated Temperature on Pisum sativum Nodule Development: I—Detailed Characteristic of Unusual Apical Senescence
Source: Int J Mol Sci. 2023 Dec 5;24(24):17144. doi: 10.3390/ijms242417144 (PMC10742560; doi:10.3390/ijms242417144)
Supplement: Supplementary file 1 [file ijms-24-17144-s001.zip › Serova_et_al_2023_supplementary_data.pdf]

SUPPLEMENTARY DATA for

Effects of elevated temperature on *Pisum sativum* nodule development: I—Detailed characteristic of unusual apical senescence

Tatiana A. Serova†, Pyotr G. Kusakin†, Anna B. Kitaeva, Elena V. Seliverstova, Artemii P. Gorshkov, Daria A. Romanyuk, Vladimir A. Zhukov, Anna V. Tsyganova, Viktor E. Tsyganov

Department of Biotechnology, All-Russia Research Institute for Agricultural Microbiology, Podbelsky Chaussee 3, 196608, Pushkin 8, Saint Petersburg, Russia

* Author for correspondence: Viktor E. Tsyganov, e-mail: vetsyganov@arriam.ru

† These authors contributed equally to this work

Figure S1. Histological and ultrastructural organization in heat-unstressed nodules of the pea (*Pisum sativum*) line SGE in 1 day of exposure to 21 °C. (A) General view. (B, D, F) Histological and (C, E, G) ultrastructural organization of nodule tissue: cells from the infection zone (B, C), nitrogen fixation zone (D, E) and individual degrading cells in the base of the nodule (F, G). I, meristem zone; II, infection zone; III, nitrogen fixation zone. ic, infected cell; uic, uninfected cell; dic, degraded infected cell; n, nucleus; id, infection droplet; b, bacterium; ba, bacteroid; jba, juvenile bacteroid; dba, degrading bacteroid; s, starch. The arrow indicates an infection thread, the triangle indicates an infection droplet. Histological sections were stained with toluidine blue. Plants were inoculated with the *Rhizobium leguminosarum* bv. *viciae* 3841 strain. Scale bars: 500 μm (A), 40 μm (B, D, F), 5 μm (C), and 1 μm (E, G).

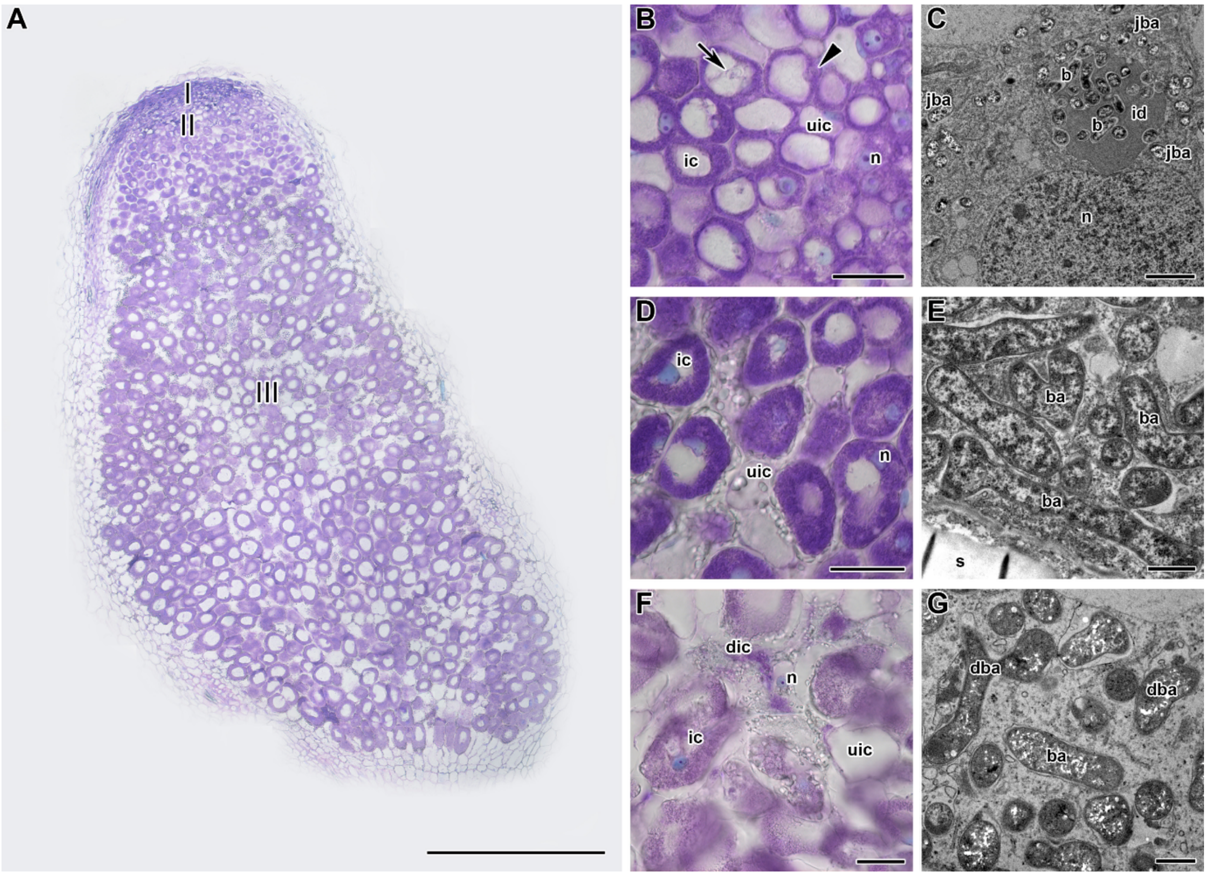

Figure S2. Histological and ultrastructural organization in heat-unstressed nodules of the pea (*Pisum sativum*) line SGE in 9 days of exposure to 21 °C. (A) General view. (B, D, F) Histological and (C, E, G) ultrastructural organization of nodule tissue: cells from the infection (B, C), nitrogen fixation (D, E) and senescence (F, G) zones. I, meristem zone; II, infection zone; III, nitrogen fixation zone; IV, senescence zone. ic, infected cell; uic, uninfected cell; dic, degraded infected cell; n, nucleus; it, infection thread; b, bacterium; ba, bacteroid; jba, juvenile bacteroid; dba, degrading bacteroid; s, starch; cw, cell wall. The triangle indicates an infection droplet. Histological sections were stained with toluidine blue. Plants were inoculated with the *Rhizobium leguminosarum* bv. *viciae* 3841 strain. Scale bars: 500 μm (A), 40 μm (B, D, F), 5 μm (G) and 1 μm (C, E).

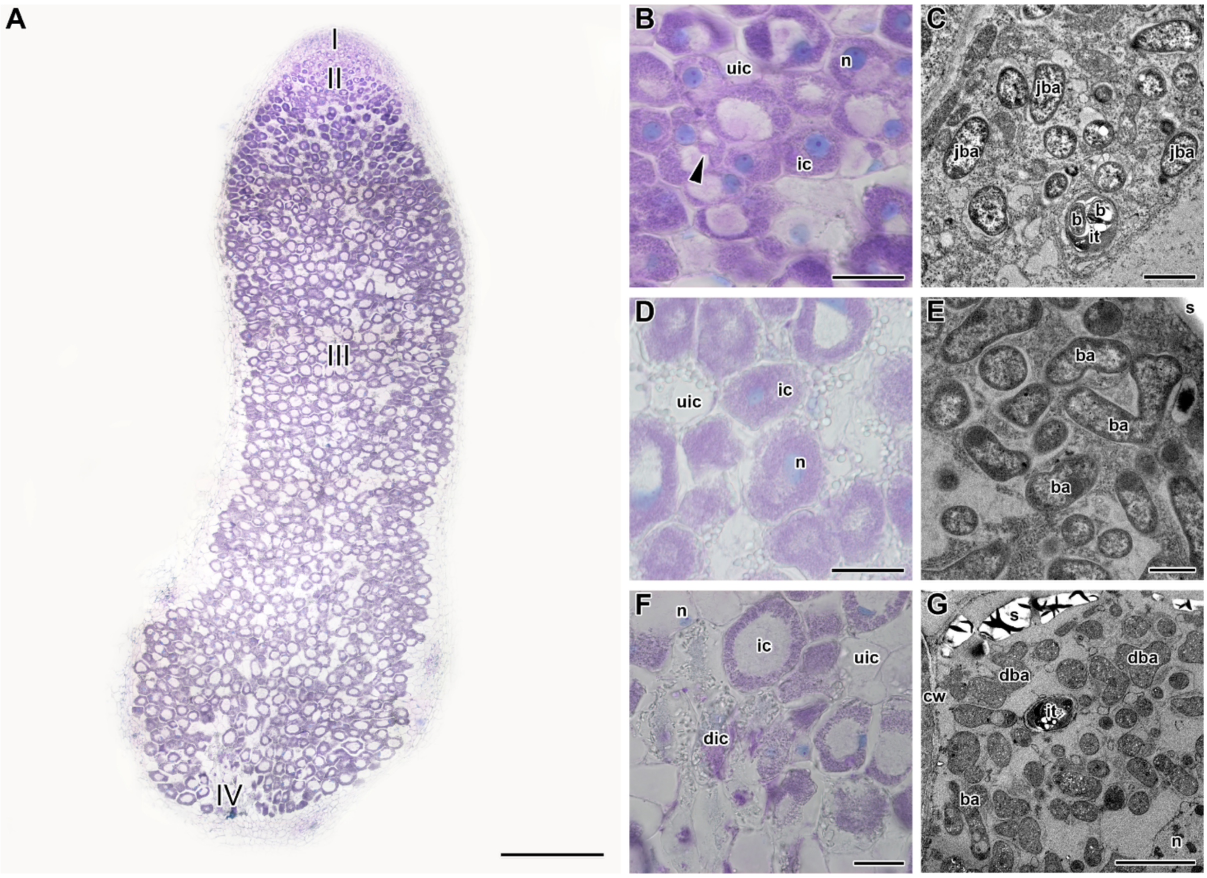

Figure S3. Heatmap of differentially expressed genes during exposure to elevated temperature. Expression changes are color-coded: upregulation in red, downregulation in blue. Each column represents one biological replicate.

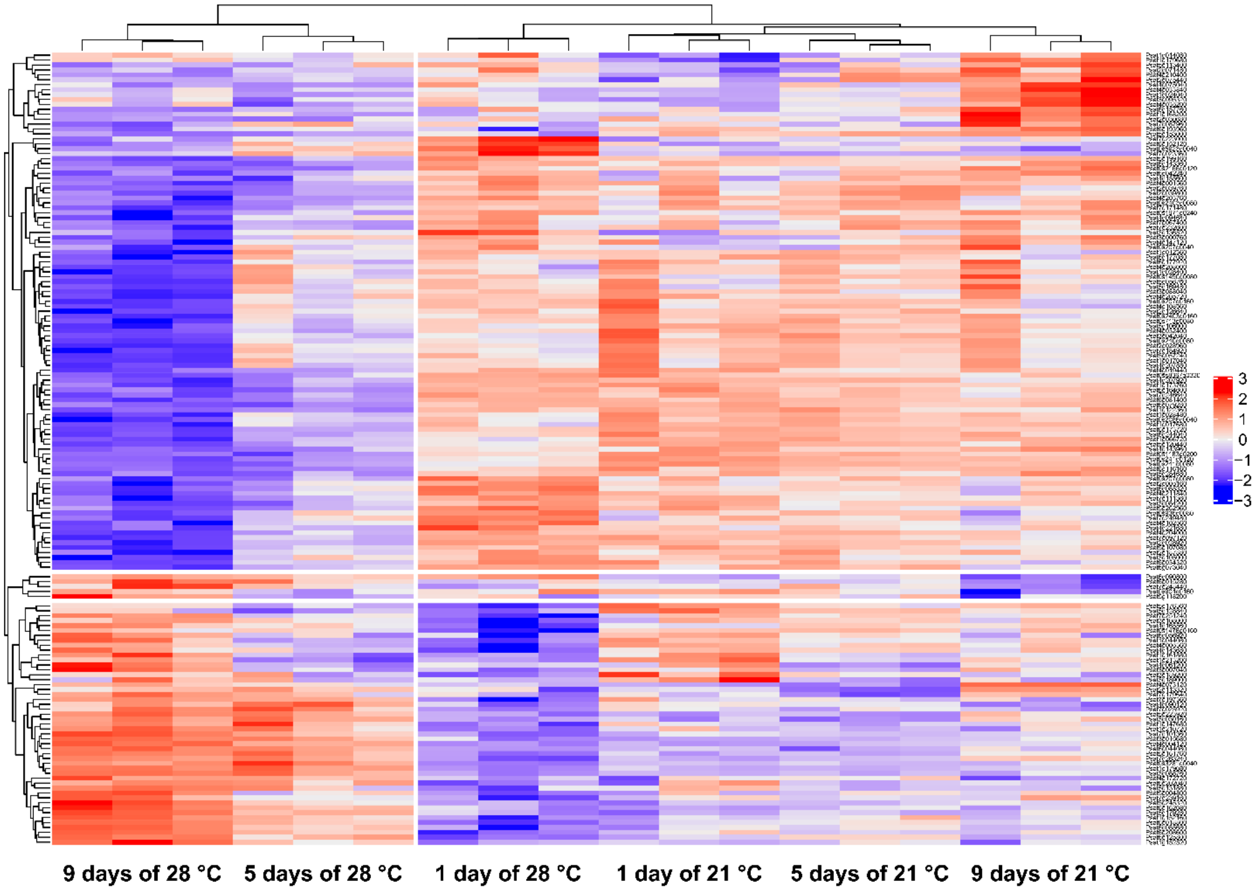

Figure S4. Expression profiles of genes demonstrating downregulation since 5 days of exposure in heat-unstressed and heat-stressed nodules of the pea (*Pisum sativum*) line SGE in 1, 5, and 9 days of exposure. Temperature conditions are color-coded: 21 °C in blue, 28 °C in red. #, number of the row in Table S2; mean 28 °C fold change (expression fold change in heat-stressed nodules between the maximum and minimum values, averaged over three replications for each time point). Gene annotation provided with the reference genome is given. Plants were inoculated with the *Rhizobium leguminosarum* bv. *viciae* 3841 strain.

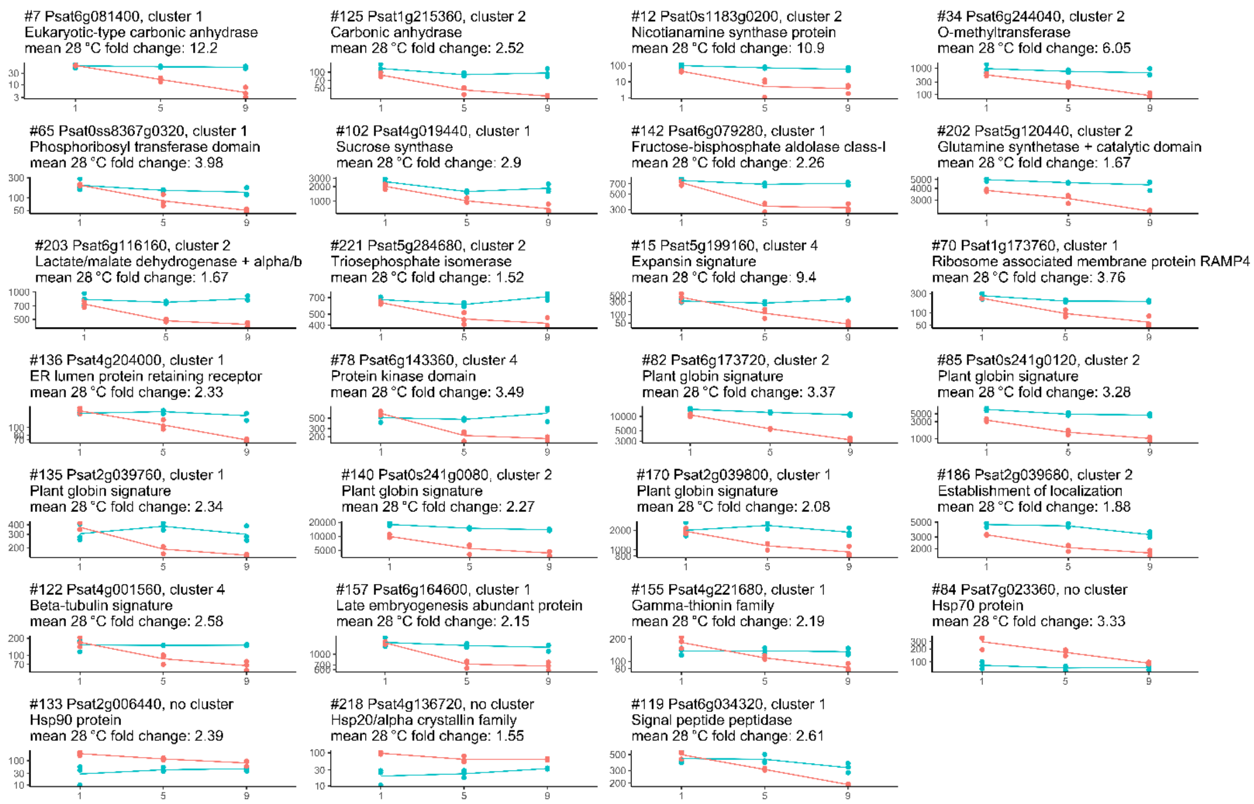

Figure S5. Expression profiles of genes demonstrating downregulation at 9 days of exposure in heat-unstressed and heat-stressed nodules of the pea (*Pisum sativum*) line SGE in 1, 5, and 9 days of exposure. Temperature conditions are color-coded: 21 °C in blue, 28 °C in red. #, number of the row in Table S2; mean 28 °C fold change (expression fold change in heat-stressed nodules between the maximum and minimum values, averaged over three replications for each time point). Gene annotation provided with reference genome is given. Plants were inoculated with the *Rhizobium leguminosarum* bv. *viciae* 3841 strain.

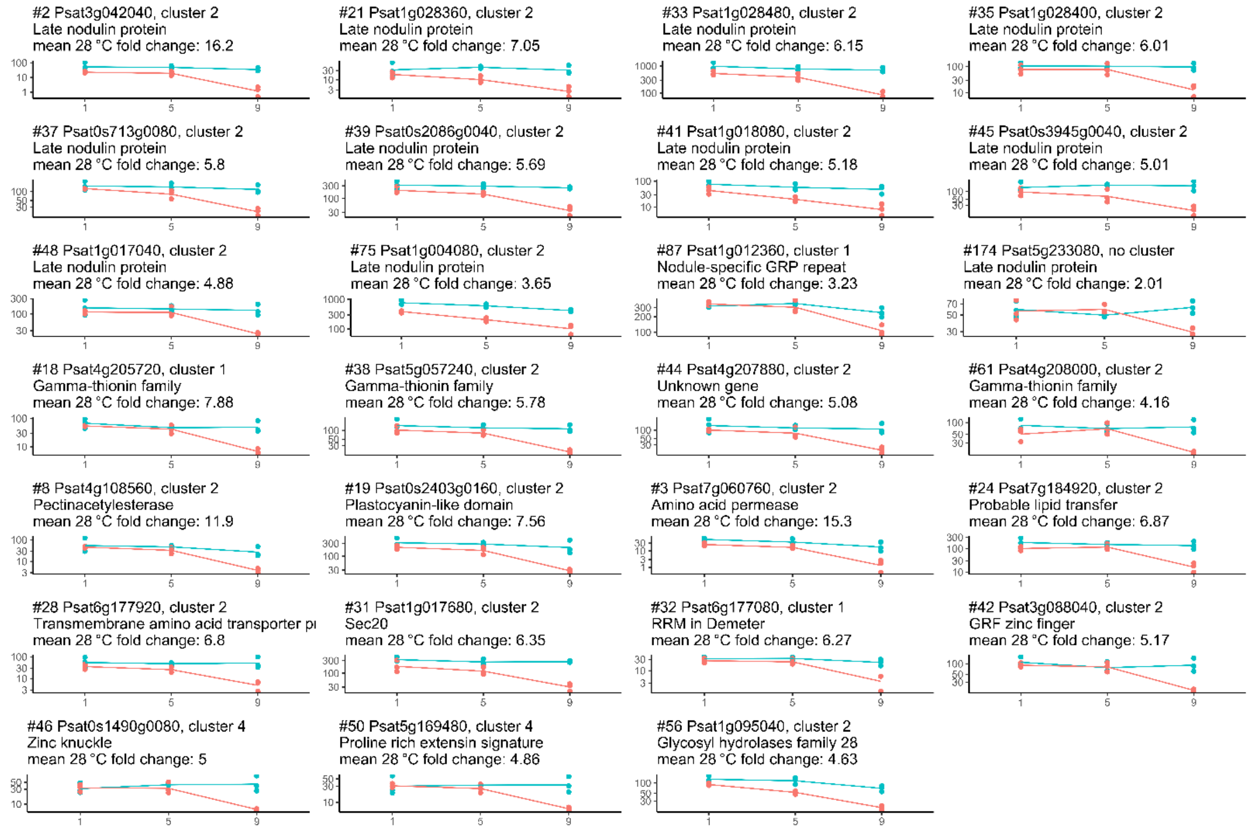

Figure S6. Expression profiles of genes demonstrating upregulation in heat-stressed compared to heat-unstressed nodules of the pea (*Pisum sativum*) line SGE in 5 and 9 days of exposure. Temperature conditions are color-coded: 21 °C in blue, 28 °C in red. #, number of the row in Table S2; mean 28 °C fold change (expression fold change in heat-stressed nodules between the maximum and minimum values, averaged over three replications for each time point). Gene annotation provided with reference genome is given. Plants were inoculated with the *Rhizobium leguminosarum* bv. *viciae* 3841 strain.

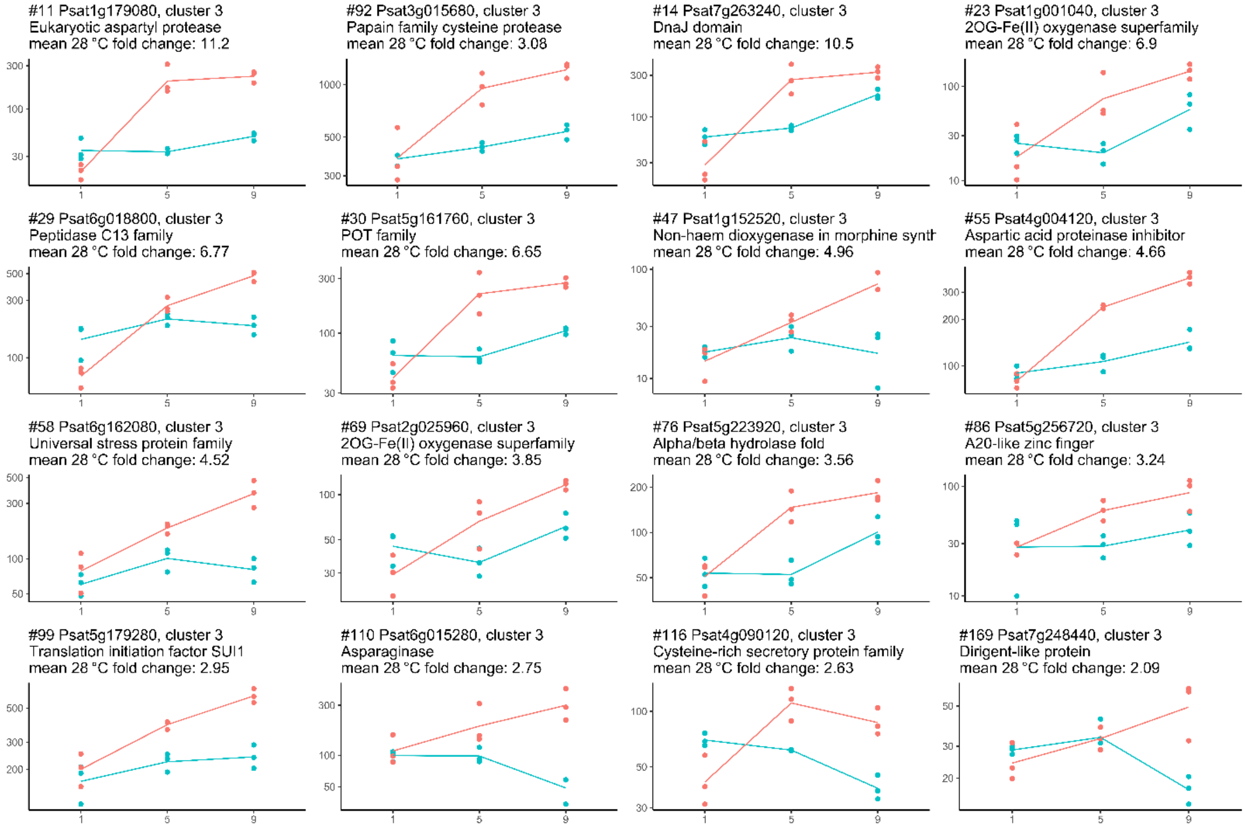

Figure S7. Expression profiles of genes downregulated in heat-stressed nodules in 1 day of exposure compared to heat-unstressed nodules, but upregulated in subsequent days. Profiles are given for heat-unstressed and heat-stressed nodules of the pea (*Pisum sativum*) line SGE in 1, 5, and 9 days of exposure. Temperature conditions are color-coded: 21 °C in blue, 28 °C in red. #, number of the row in Table S2; mean 28 °C fold change (expression fold change in heat-stressed nodules between the maximum and minimum values, averaged over three replications for each time point). Gene annotation provided with reference genome is given. Plants were inoculated with the *Rhizobium leguminosarum* bv. *viciae* 3841 strain.

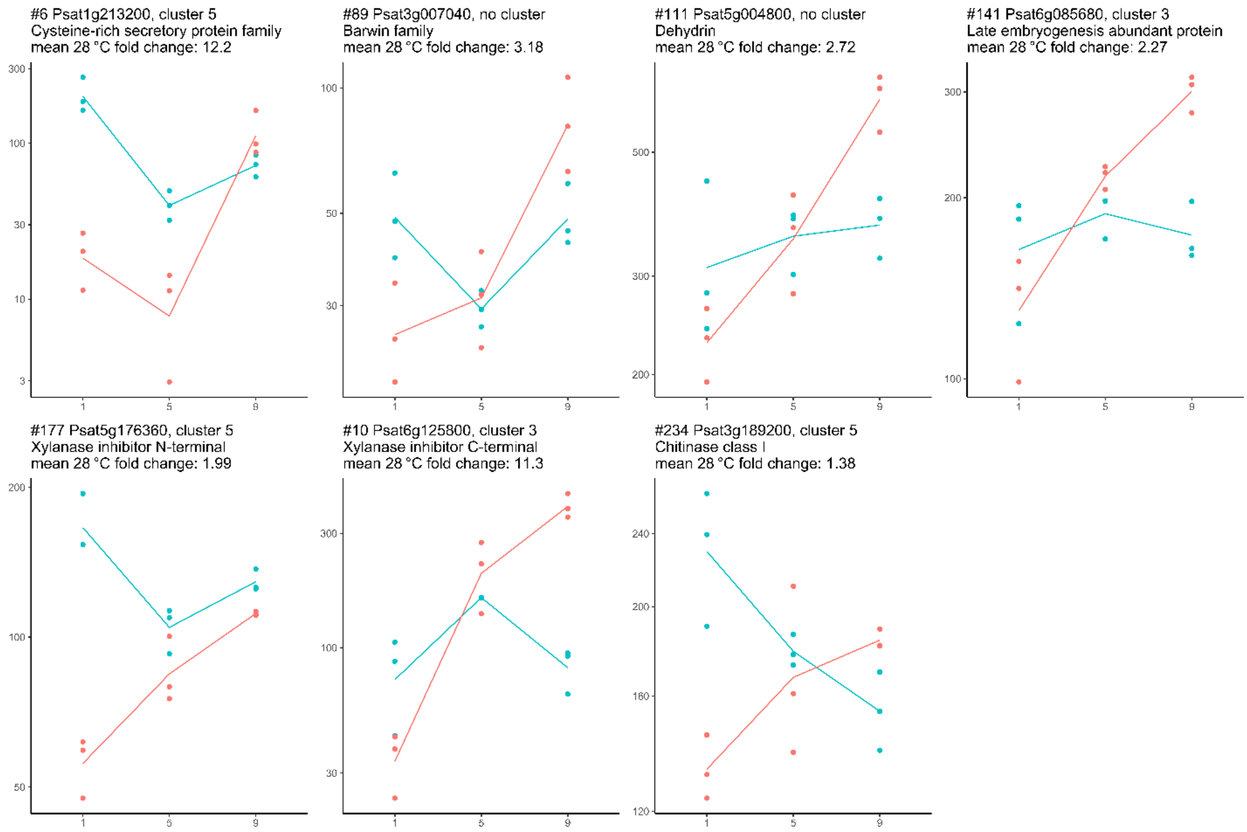

Figure S8. Nodules of the pea (*Pisum sativum*) line SGE exposed to elevated temperature during the days and to the optimal one during the night. (A) Whole nodule, (B) longitudinally cut nodule. Plants were inoculated with the *Rhizobium leguminosarum* bv. *viciae* 3841 strain. Scale bars are 1 mm.

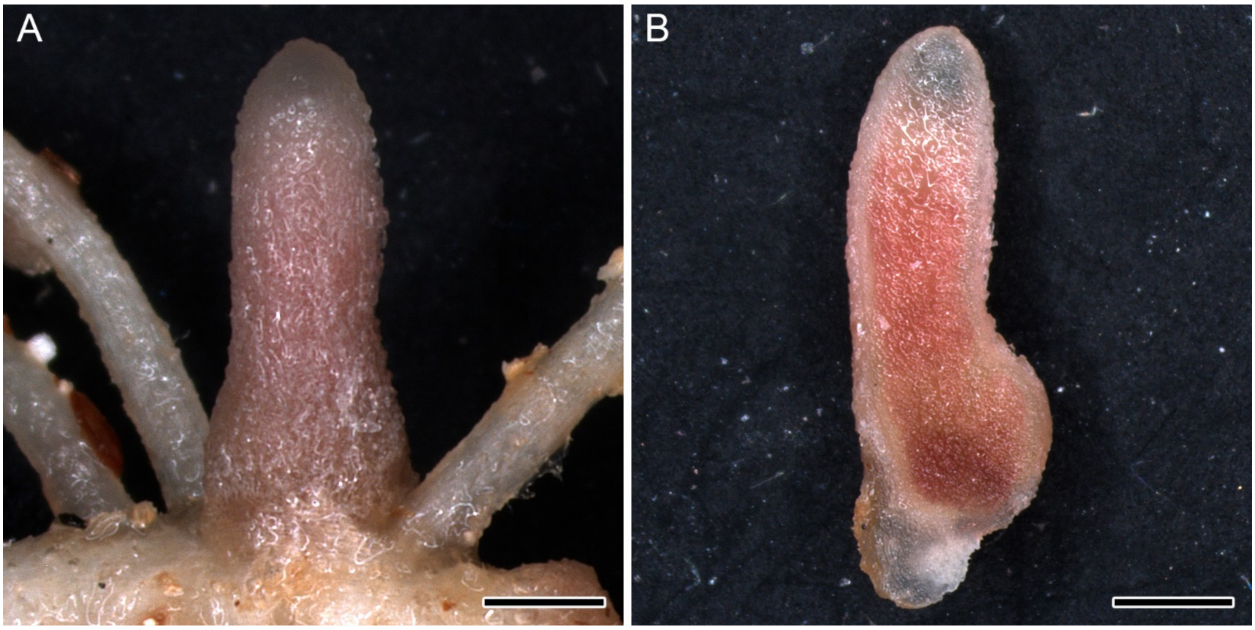

Figure S9. Scheme of the experiment on the effect of elevated temperature (28 °C) on the functioning of the pea (*Pisum sativum*) nodules. WAI, weeks after inoculation with *Rhizobium leguminosarum* bv. *viciae* 3841.

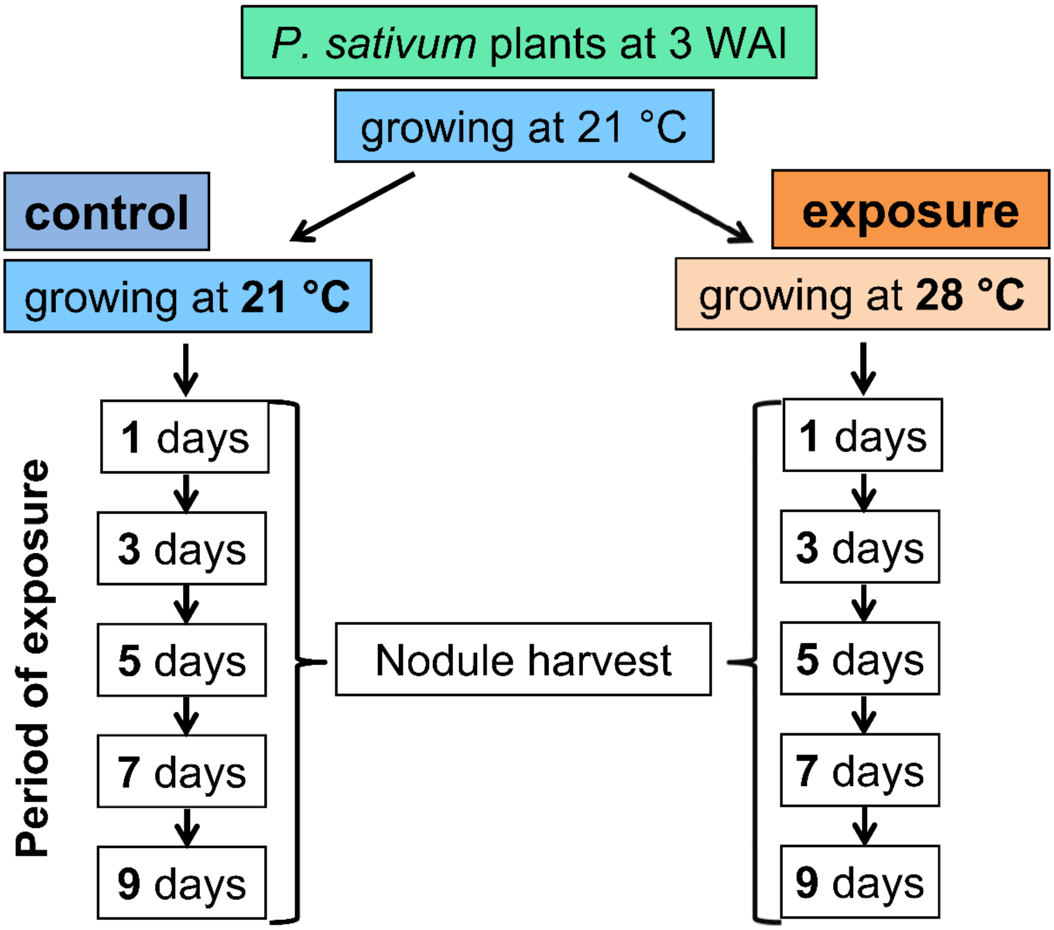

Figure S10. Scheme of the experiment on the nodule recovery of the pea (*Pisum sativum*) line SGE after transferring back from elevated temperature (28 °C) to the optimum one (21 °C). WAI, weeks after inoculation with *Rhizobium leguminosarum* bv. *viciae* 3841.

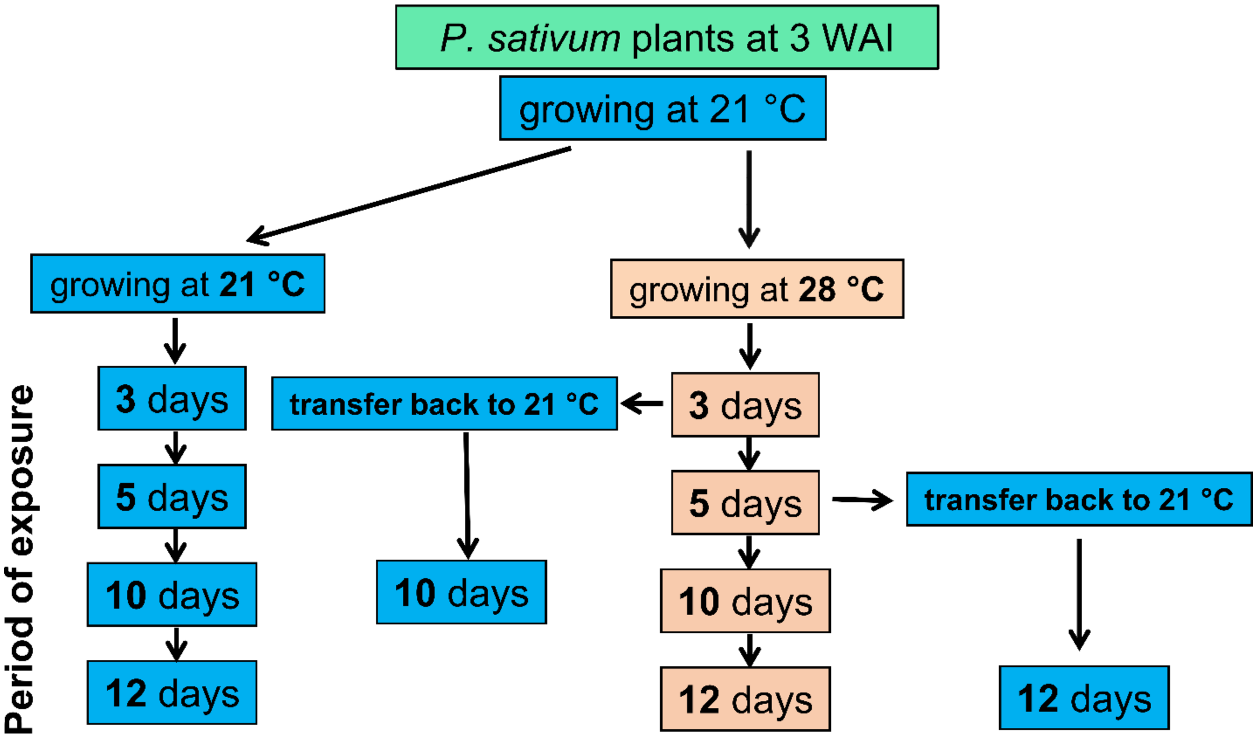

Table S1. Description of primer sequences for expression analysis of *Pisum sativum* selected genes.

Gene	Description	Accession no.	Primer sequence (5′–3′)	Amplicon size (bp)	Reference
PsCyp15a	Cysteine protease 15a	X54358.1	^{1107}GTAGCTGCAGCTCAATCCAACC<br>^{1304}CATCACCACAGTAACAGCAAGACA	222	[34]
Ps26S AAA-ATPase***	26S proteasome AAA-ATPase subunit	PsCam000968*	^{8} AATGGGGTCACGTAACATAGCG<br>^{105} GATGGAAGGGGTGAAGGTTAGG	119	This study
PsATB2	bZIP transcription factor	69046**	^{104}GAGACGGTCTCGGATGAGGAAA<br>^{351}TCAGAGGGTTGAAGAAGAAGAAGC	272	[34]
PsHsr203J	Hypersensitivity response marker	AB026296.1	^{175}CACTACCACCAACGACAACTTCA<br>^{214}GGCGTTTTCTCCGGTAGGTAT	60	[36, 93]

PsACS2	ACC synthase 2	AF016459.1	^{1495}GGCATAGTAATTTGAGGTTGAGCC<br>^{1695}GCCCCAACATTTAAAGGACCTATTA	226	[34]
PsACO1	ACC oxidase 1	M98357.1	^{862}TACATGGGACTCAAGTTCCAAGCT<br>^{995}GCACAATCTTAAAACACCAACCAAA	159	[34]
PsNCED2	9-cis-epoxycarotenoid dioxygenase 2	AB080192.1	^{96}GAACCAATCTTCTCCACTATGGCA<br>^{232}AAGGGAGTGTTGTTTGTAGCGAAC	163	[53]
PsAO3	Aldehyde oxidase 3	EF491600.1	^{4386}TTATAGGACACAGGCTAGCTCAGCA<br>^{4487}TGACACAAGCTTATTCAGCATGACA	127	[34]
PsGA20ox1	GA 20-oxidase 1	U70471.1	^{874}CATTCCATTAGGCCAAATTTCAAT<br>^{945}CTGCCCTATGTAAACAACTCTTGTATCT	100	[94]
PsGA2ox1	GA 2-β-hydroxilase 1	AF056935.1	^{1009}GCTGCCACTTAATATTGGAGGATC<br>^{1236}GAGTGTTGATGCAAAAGGGGAA	250	[34]

PsLoxN1	Lipoxygenase 1	U84198.1	[71]ACATGGCAACAAAGGTGTTTGG<br>[162]TTTACCGATGGACGTTATAGCG	113	This study
PsHS TF B-3*	Heat stress transcription factor B-3	PsCam039062*	[3]CTTGCGTTTCTCATGAACTGTGG<br>[243]GTCTTCAACAAGAAAGGCGATGG	263	This study
PsHSP22	Heat shock protein 22 kDa	X86222	[35]TCTCTCCTTCAAATACAACCGCC<br>[121]GAAGCCATTGAGATTGCGGG	106	This study
PsHSP17.9	Heat shock protein 17.9 kDa	M33900	[20]GGTACTGGACGAAGAACCAATGC<br>[117]AAAAGCTGCCGTCTCGTTGG	117	This study
PsGSH1	γ-glutamylcysteine synthetase	AF128455.1	[203]CTCCTCCGCCGCATAACTTC<br>[373]GGCGAGATAATCGATGAGATCCTG	194	[37]

PsGSHS	Glutathione synthetase	AF231137.1	[28]GCCGCTGATTTTCGTTCCACTA<br>[193]CGACGTCGACGGTTTGTTTACC	187	[37]
PsGapC1	Glyceraldehyde-3-phosphate dehydrogenase	L07500.1	[222]AAGAACGACGAACTCACCG<br>[389]TTGGCACCACCCTTCAAATG	188	[95]

* contig sequence for *P. sativum* in the database https://urgi.versailles.inra.fr/

** contig sequence for *P. sativum* in the database https://www.coolseasonfoodlegume.org/

*** gene symbols were created by the authors based on the annotation of contig sequence
